# Migraine phenotypes defined by disability, pain topography and autonomic features: association with serum vitamin D status

**DOI:** 10.3389/fneur.2026.1822813

**Published:** 2026-07-08

**Authors:** Majed Mohammad Alabdali

**Affiliations:** Department of Neurology, College of Medicine, Imam Abdulrahman Bin Faisal University, Dammam, Saudi Arabia

**Keywords:** autonomic symptoms, cluster analysis, disability, K-means, migraine, phenotype, vitamin D

## Abstract

Migraine presents with substantial clinical heterogeneity, yet studies examining the link between vitamin D and migraine typically treat patients as a uniform group. Whether distinct clinical phenotypes harbor differential vitamin D levels remains unexplored. This study aimed to identify data-driven migraine phenotypes using cluster analysis and to compare serum 25-hydroxyvitamin D levels within this clinical population, across these phenotypes. This retrospective chart review included 95 migraine patients with complete data on HIT-6, MIDAS grade, attack severity, disease duration, pain lateralization, autonomic symptoms, and serum vitamin D. K-means clustering (k = 4) was applied to sixteen standardized clinical features; cluster stability was quantified by bootstrap resampling, and findings were corroborated using cluster-independent regression and alternative clustering algorithms suited to mixed data. Vitamin D was compared across clusters using Kruskal–Wallis and ANOVA tests. Logistic regression assessed whether vitamin D predicted severe phenotype membership adjusting for age, sex, and duration. Four phenotypes emerged: a left-sided aura-rich cluster (*n* = 11) with the lowest vitamin D (median 9.54 ng/mL); a right-sided moderate cluster (*n* = 26); a bilateral high-disability cluster (*n* = 28) with prominent nausea and photo/phonophobia; and a bilateral lower-disability cluster (*n* = 30) marked by dizziness and palpitations. Vitamin D was broadly deficient (mean 17.01 ± 9.71 ng/mL; 67.4% below 20 ng/mL), at a rate statistically indistinguishable from contemporary Saudi general-population estimates (~63–65%; binomial *p* = 0.40), indicating that the deficiency is a background population phenomenon rather than a migraine-specific finding, but differences across clusters did not reach significance (Kruskal–Wallis *p* = 0.153). Female sex was the only significant predictor of severe phenotype membership (OR 3.37, 95% CI 1.20–9.46, *p* = 0.021); this effect, and the vitamin D null, were reproduced in cluster-free continuous models and were invariant to the clustering algorithm used. Clinically interpretable migraine phenotypes can be derived from routine variables, although they represent a descriptive partition of a continuous phenotypic space rather than discrete biological subtypes. Although vitamin D deficiency was highly prevalent, it did not differ significantly across phenotypes, though one aura-rich subgroup showed notably low levels warranting investigation in larger cohorts that include non-migraine comparison groups.

## 1 Introduction

Migraine is among the most prevalent neurological disorders globally, affecting over one billion people and ranking as the second leading cause of years lived with disability (1). Its burden falls disproportionately on women of reproductive age and carries substantial societal costs through lost productivity and healthcare utilization (2). Despite this, the condition remains underdiagnosed in many clinical settings. Under a single diagnostic label, migraine encompasses considerable variability, from infrequent moderate episodes to severe unilateral attacks accompanied by prominent autonomic disturbances and marked functional disability. Classical systems such as the International Classification of Headache Disorders distinguish migraine with and without aura (3), but this binary categorization fails to capture the full phenotypic spectrum encountered in practice.

In recent years, data-driven approaches including cluster analysis have been applied to identify naturally occurring migraine subgroups, revealing phenotypes that conventional criteria do not delineate (4). Such efforts hold promise for understanding the biological heterogeneity of migraine and guiding personalized management. In parallel, vitamin D has attracted growing interest as a modifiable factor in migraine pathophysiology. Observational studies have reported that patients with migraine tend to have lower serum 25-hydroxyvitamin D concentrations compared with non-migraine controls, and some evidence links greater deficiency with higher headache frequency (5). A large Korean study found vitamin D deficiency in 77.1% of migraineurs, with monthly headache frequency 1.2 times higher in deficient patients compared with those with adequate levels (6).

Biologically, vitamin D participates in immune regulation, modulation of inflammatory cytokines, and serotonin synthesis, all pathways implicated in migraine pathogenesis (7). A meta-analysis of randomized controlled trials reported that vitamin D supplementation significantly reduced headache attack frequency and MIDAS disability scores (8). However, these studies have invariably treated migraine as a homogeneous condition without considering whether specific phenotypic subgroups harbor more severe deficiency. To our knowledge, no published study has examined whether data-driven migraine phenotypes (defined by disability, pain topography, and autonomic features) differ in their vitamin D status. The present study used unsupervised cluster analysis on routine clinical data to derive migraine phenotypes and compared serum 25-hydroxyvitamin D levels across these subgroups. Importantly, the question addressed here is one of within-disease stratification, whether vitamin D status tracks with clinical phenotype among patients who already carry a migraine diagnosis, rather than a case control question of whether migraineurs differ from non-migraineurs. The latter has been examined extensively elsewhere (5, 6), whereas the former, to our knowledge, has not. This distinction frames both our design and the interpretation of our findings.

## 2 Methods

This study was conducted and reported in accordance with the Strengthening the Reporting of Observational Studies in Epidemiology (STROBE) guidelines for cross-sectional studies (9).

### 2.1 Study design and participants

This retrospective chart review included patients with clinician-diagnosed migraine attending a neurology outpatient clinic. This study was reviewed and approved by the Institutional Review Board, Imam Abdulrahman Bin Faisal University, Saudi Arabia (IRB approval number: IRB-2025-01-0901). Patients were included if they had complete records for HIT-6 score, MIDAS disability grade, self-reported attack severity (0–10 scale), disease duration category, pain lateralization, documented symptoms, and serum 25-hydroxyvitamin D measured within three months of clinical assessment. Of 104 consecutive records screened, 95 met all criteria and formed the analysis sample.

### 2.2 Variables

Demographic variables included age and sex. Clinical variables comprised HIT-6 score, MIDAS grade (ordinal I–IV), severity, duration (ordinal: <6, 6–12, 12–24, >24 months), and pain lateralization (right, left, bilateral/global or unclear). Symptoms—aura, photophobia, phonophobia, nausea/vomiting, dizziness/vertigo, palpitations, numbness, and throbbing—were extracted from free-text clinical notes using pattern matching with accommodation for misspellings. To validate this automated extraction, a 20% random sample of records was reviewed manually by the author, yielding a Cohen’s kappa of 0.91, indicating excellent agreement. Aura was coded separately from autonomic symptoms, as aura reflects cortical rather than autonomic mechanisms. Serum 25-hydroxyvitamin D was categorized as deficient (<10), insufficient (10–<20), sub-optimal (20–<30), and sufficient (≥30 ng/mL) following established thresholds (10).

### 2.3 Statistical analysis

All sixteen clinical features were z-score normalized prior to clustering to ensure equal weighting across variables with different scales. K-means clustering with k-means++ initialization was applied to the standardized features (HIT-6, MIDAS, severity, duration, four lateralization dummies, two topography indicators, six symptom flags), using 100 random restarts to ensure stability of the solution. The number of clusters was selected using silhouette scores and clinical interpretability. Silhouette scores for k = 2 through 7 were as follows: k = 2 (0.176), k = 3 (0.159), k = 4 (0.168), k = 5 (0.173), k = 6 (0.155), k = 7 (0.132). Although k = 5 yielded a marginally higher silhouette score (0.173 vs. 0.168), it produced a cluster containing only 6 patients, deemed too small for reliable characterization. Therefore, k = 4 was selected as the optimal balance between cluster separation and clinical interpretability, consistent with recommendations for exploratory clinical clustering (4).

Vitamin D was compared across clusters using Kruskal–Wallis and one-way ANOVA tests, with effect sizes reported as epsilon-squared (ε^2^) and eta-squared (η^2^), respectively. The vitamin D category distribution was assessed with the chi-square test. Logistic regression estimated the association between vitamin D and membership in a composite “severe phenotype” (top-ranked clusters by a composite severity score, defined as the cluster-level average of individual mean z-scores across standardized HIT-6, MIDAS, and attack severity), adjusting for age, sex, and duration. Analyses used Python 3.12 (scikit-learn, SciPy); significance was set at *p* < 0.05.

K-means was selected as the primary algorithm for its transparency, reproducibility, and wide use in exploratory clinical phenotyping; because k-means assumes Euclidean geometry that is strictly appropriate only for continuous inputs, and our feature set combined continuous, ordinal, and binary variables, we explicitly tested whether the partition and, more importantly, the substantive conclusions were dependent on this choice. Three complementary validation steps were therefore prespecified. First, cluster stability was quantified using non-parametric bootstrap resampling (B = 500), computing for each cluster the mean Jaccard coefficient between the original cluster and its closest match in each resampled solution, following the framework of Hennig, in which mean Jaccard values below 0.60 indicate that a cluster should be regarded as a descriptive grouping rather than a stable, reproducible entity. Second, the analysis was repeated using two algorithms better suited to mixed-type data: k-prototypes (which handles continuous and categorical variables jointly) and agglomerative hierarchical clustering on a Gower dissimilarity matrix; agreement with the k-means solution was summarized using the adjusted Rand index (ARI), and the primary vitamin D comparison was re-run within each alternative partition. Third, to ensure that the principal inferences did not depend on the clustering step at all, we conducted cluster-free analyses on the raw variables, relating serum vitamin D to a continuous composite severity score (mean of standardized HIT-6, MIDAS, and attack severity) using Spearman correlation and multivariable linear regression adjusted for age, sex, and duration.

Because no concurrent non-migraine control group was available, the cohort-wide prevalence of deficiency (25(OH)D < 20 ng/mL) was benchmarked against contemporary Saudi general-population estimates using a one-sample exact binomial test, to assess whether deficiency in this migraine sample exceeded the high background rate documented at the population level.

## 3 Results

### 3.1 Sample characteristics

Of 104 records screened, 95 met inclusion criteria. Baseline demographic and clinical characteristics of the full cohort are presented in Table 1. Mean age was 43.32 ± 14.10 years and 67 (70.5%) were female. Mean HIT-6 was 59.83 ± 9.21 and mean severity 7.93 ± 1.67. MIDAS grades were distributed as I = 6, II = 33, III = 35, IV = 21. Bilateral headache was the most common pattern (54.7%), followed by right-sided (28.4%). The most frequent symptoms were aura (41.1%), nausea/vomiting (32.6%), and photophobia (28.4%); 77.9% had at least one autonomic symptom excluding aura. Mean serum vitamin D was 17.01 ± 9.71 ng/mL (median 13.60, IQR 9.25–24.15), with 67.4% below 20 ng/mL and only 12.6% sufficient (≥30 ng/mL). This cohort wide deficiency rate (67.4% below 20 ng/mL) did not differ significantly from contemporary Saudi general-population estimates of approximately 63–65% in comparable adult cohorts (one-sample exact binomial *p* = 0.40 against 63%; *p* = 0.67 against 65%) (11, 12), indicating that the high prevalence of deficiency observed here is consistent with the documented background population rate rather than representing a migraine-specific excess. Serum 25(OH)D did not differ between the sexes (mean 17.07 ng/mL in males vs. 16.98 ng/mL in females; *p* = 0.98), so the cohort-wide deficiency is not an artifact of the female predominance of the sample.

**Table 1:** Baseline demographic and clinical characteristics of the study cohort (*N* = 95).

Variable	Full cohort (*N* = 95)
Age, years, mean ± SD	43.32 ± 14.10
Female sex, *n* (%)	67 (70.5)
HIT-6 score, mean ± SD	59.83 ± 9.21
MIDAS grade, *n* (%)	
Grade I	6 (6.3)
Grade II	33 (34.7)
Grade III	35 (36.8)
Grade IV	21 (22.1)
Attack severity (0–10), mean ± SD	7.93 ± 1.67
Pain lateralization, *n* (%)	
Right-sided	27 (28.4)
Left-sided	16 (16.8)
Bilateral/global	52 (54.7)
Symptoms, *n* (%)	
Aura	39 (41.1)
Nausea/vomiting	31 (32.6)
Photophobia	27 (28.4)
Phonophobia	22 (23.2)
Dizziness/vertigo	20 (21.1)
Palpitations	14 (14.7)
Numbness	12 (12.6)
Any autonomic symptom (excl. aura)	74 (77.9)
Serum 25(OH)D, ng/mL, mean ± SD	17.01 ± 9.71
Median (IQR)	13.60 (9.25–24.15)
Vitamin D category, *n* (%)	
Deficient (<10 ng/mL)	28 (29.5)
Insufficient (10–<20 ng/mL)	36 (37.9)
Sub-optimal (20–<30 ng/mL)	19 (20.0)
Sufficient (≥30 ng/mL)	12 (12.6)

SD, standard deviation; HIT-6, Headache Impact Test-6; MIDAS, Migraine Disability Assessment; 25(OH)D, 25-hydroxyvitamin D; IQR, interquartile range.

### 3.2 Cluster analysis

**Figure 1:**
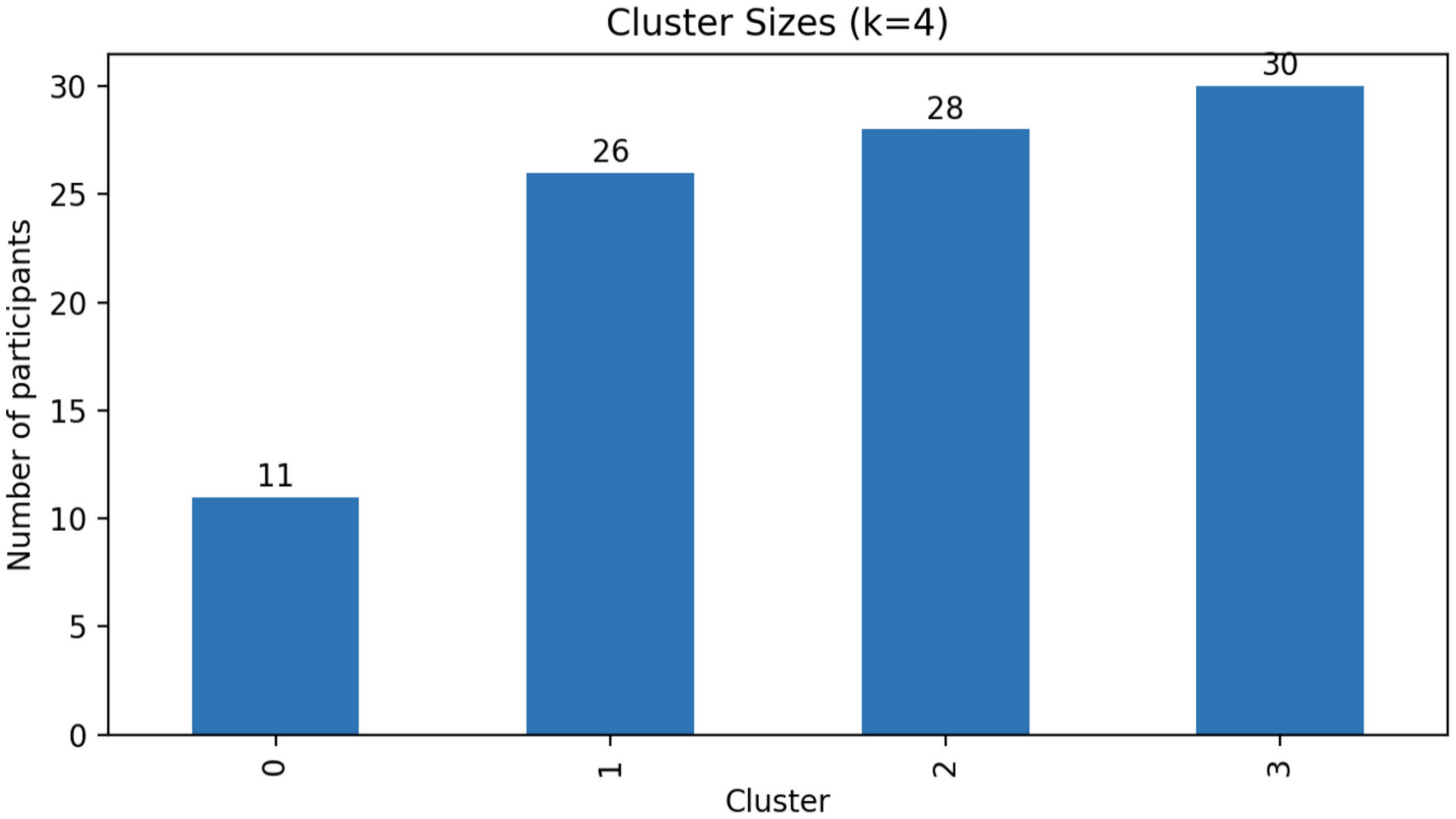
Patient distribution across the four identified clusters.

K-means was evaluated for k = 2 through 7. Silhouette scores ranged from 0.132 to 0.176, indicating modest but not unusual cluster boundaries for mixed clinical data. K = 4 was selected as the optimal trade-off, yielding a silhouette score of 0.168 (Supplementary Figures S1, S2). The four clusters are profiled in Table 2 and Figure 1. Consistent with these modest silhouette values, bootstrap resampling (B = 500) yielded a mean Jaccard stability coefficient of 0.48 across the four clusters (range 0.43–0.53), confirming that the cluster boundaries are not sharply reproducible and that the phenotypes are best interpreted as a descriptive partition of a continuous phenotypic space rather than as discrete, reproducible disease subtypes. The robustness of the study’s substantive conclusions to this instability is addressed directly in Section 3.6.

**Table 2:** Clinical profiles of the four migraine phenotypes identified by k-means clustering (k = 4).

Cluster	*n*	Pain side	HIT-6	MIDAS	Severity	Aura%	Auto%	VitD mean	VitD med.
0	11	Left	61.4	2.91	7.27	54.5	72.7	12.55	9.54
1	26	Right	58.4	2.58	7.58	38.5	73.1	15.38	14.20
2	28	Bilateral	64.7	3.32	9.11	28.6	92.9	18.99	14.95
3	30	Bilateral	56.0	2.30	7.37	50.0	70.0	18.20	13.40

Auto% = any autonomic symptom excluding aura. VitD in ng/mL. MIDAS grade ordinal (I–IV = 1–4).

Cluster 0 (*n* = 11) comprised exclusively left-sided pain with the highest aura prevalence (54.5%) and the lowest vitamin D (mean 12.55, median 9.54 ng/mL). Cluster 1 (*n* = 26) had exclusively right-sided pain with moderate disability scores. Cluster 2 (*n* = 28) was the most severe by composite score (HIT-6 64.7, severity 9.11), bilateral, with the highest photophobia (50.0%), nausea (64.3%), and autonomic burden (92.9%); 85.7% were female. Cluster 3 (*n* = 30) was the mildest, bilateral, distinguished by dizziness (36.7%) and palpitations (26.7%), with the only balanced sex ratio (50% female).

### 3.3 Vitamin D across phenotypes

**Figure 2:**
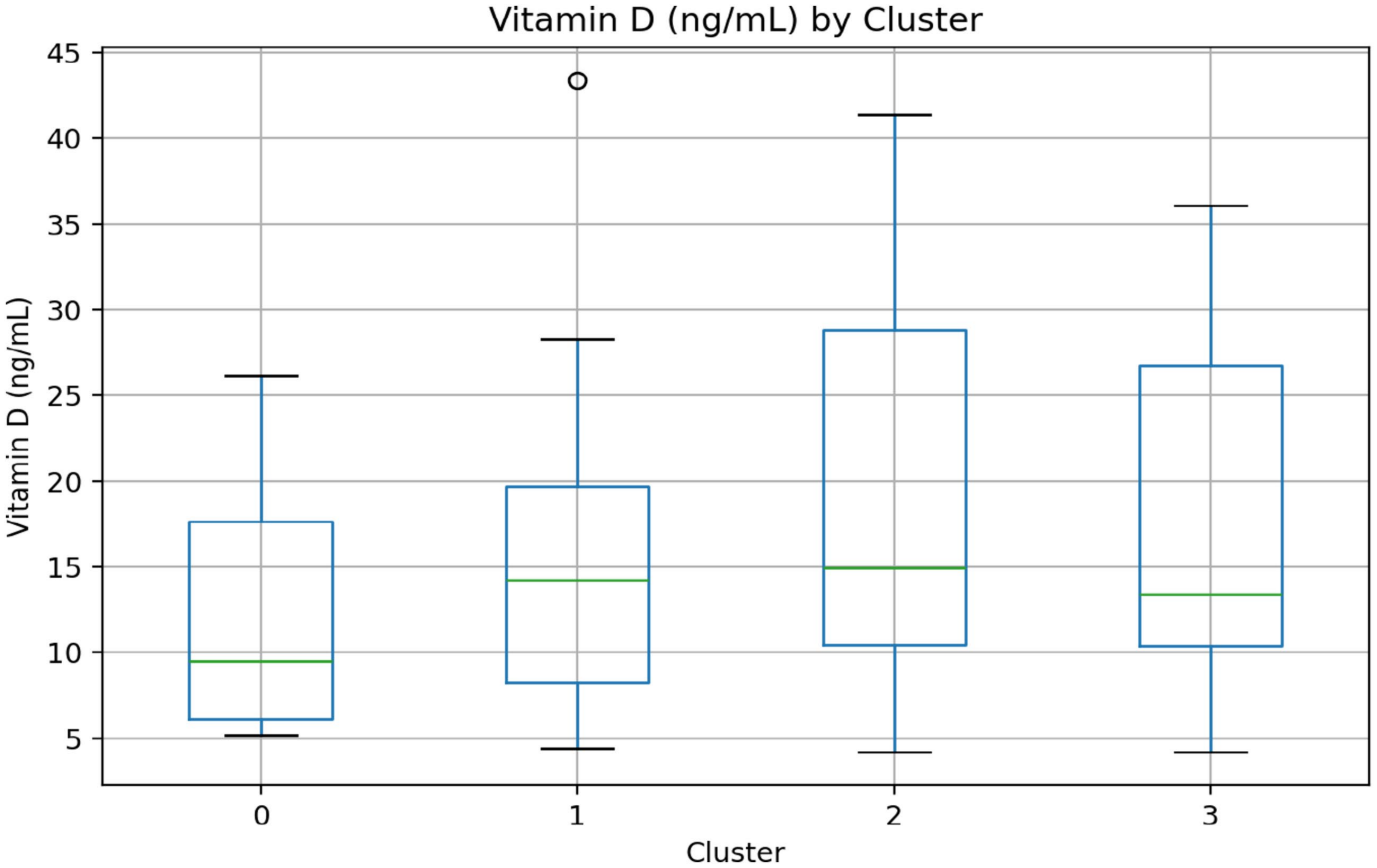
Serum vitamin D distribution by cluster. Cluster 0 shows a lower distribution, though overall differences were not statistically significant (Kruskal–Wallis *p* = 0.153).

Despite descriptively lower levels in Cluster 0, formal testing showed no significant variation (Kruskal–Wallis H = 5.27, *p* = 0.153, ε^2^ = 0.025; ANOVA *F* = 1.58, *p* = 0.199, η^2^ = 0.049). The distribution of vitamin D categories across clusters was assessed using the Fisher–Freeman–Halton exact test, given that 7 of 16 contingency cells had expected counts below 5, rendering the chi-square test unreliable. The exact test yielded *p* = 0.371, confirming no significant association between vitamin D category and cluster membership (Figure 2).

### 3.4 Logistic regression

Clusters 2 and 0 (*n* = 39), ranking highest on the severity composite, were designated the “severe phenotype.” Vitamin D showed no association with severe phenotype membership (OR 1.00, 95% CI 0.96–1.05, *p* = 0.986). Female sex was the only significant predictor (OR 3.37, 95% CI 1.20–9.46, *p* = 0.021). Duration showed a non-significant trend (OR 1.58, *p* = 0.126) (Table 3).

**Table 3:** Adjusted logistic regression: predictors of severe phenotype membership.

Predictor	OR	95% CI	*p*-value
Vitamin D (per ng/mL)	1.00	0.96–1.05	0.986
Age (per year)	1.00	0.97–1.03	0.988
**Female sex**	**3.37**	**1.20–9.46**	0.021*
Duration (ordinal step)	1.58	0.88–2.83	0.126

Bold values indicate statisticalsignificance (*p* < 0.05).

### 3.5 Sensitivity analysis

As a sensitivity analysis, Cluster 2 alone (*n* = 28) was designated as the severe group. In this model, vitamin D showed no significant association with severe phenotype membership (OR 1.02, 95% CI 0.97–1.07, *p* = 0.478). Female sex remained the only significant predictor (OR 3.89, 95% CI 1.25–12.12, *p* = 0.019), reinforcing the robustness of this finding across different severe-group definitions.

### 3.6 Robustness of conclusions to clustering method and to clustering itself

Because the modest silhouette values and bootstrap Jaccard coefficients (Section 3.2) indicated that the cluster boundaries are not strongly reproducible, we tested whether the study’s two principal conclusions (i) that serum vitamin D does not differ meaningfully with migraine phenotype, and (ii) that female sex is the dominant correlate of severe phenotype depended on the clustering algorithm or on the clustering step at all.

Reclustering the same features with k-prototypes, which is designed for combined continuous and categorical variables, produced a partition that, as expected from the instability above, only partially overlapped the k-means solution (adjusted Rand index 0.17). Crucially, however, the vitamin D result was unchanged: serum 25(OH)D did not differ across the k-prototypes clusters (Kruskal–Wallis *p* = 0.79). Agglomerative hierarchical clustering on a Gower dissimilarity matrix, a metric explicitly appropriate for mixed-type data, yielded a higher silhouette coefficient than k-means (0.22 vs. 0.16) while again showing no significant between-cluster difference in vitamin D. Thus, the absence of a phenotype–vitamin D association is invariant to the clustering method, even though the specific cluster memberships are not.

**Figure 3:**
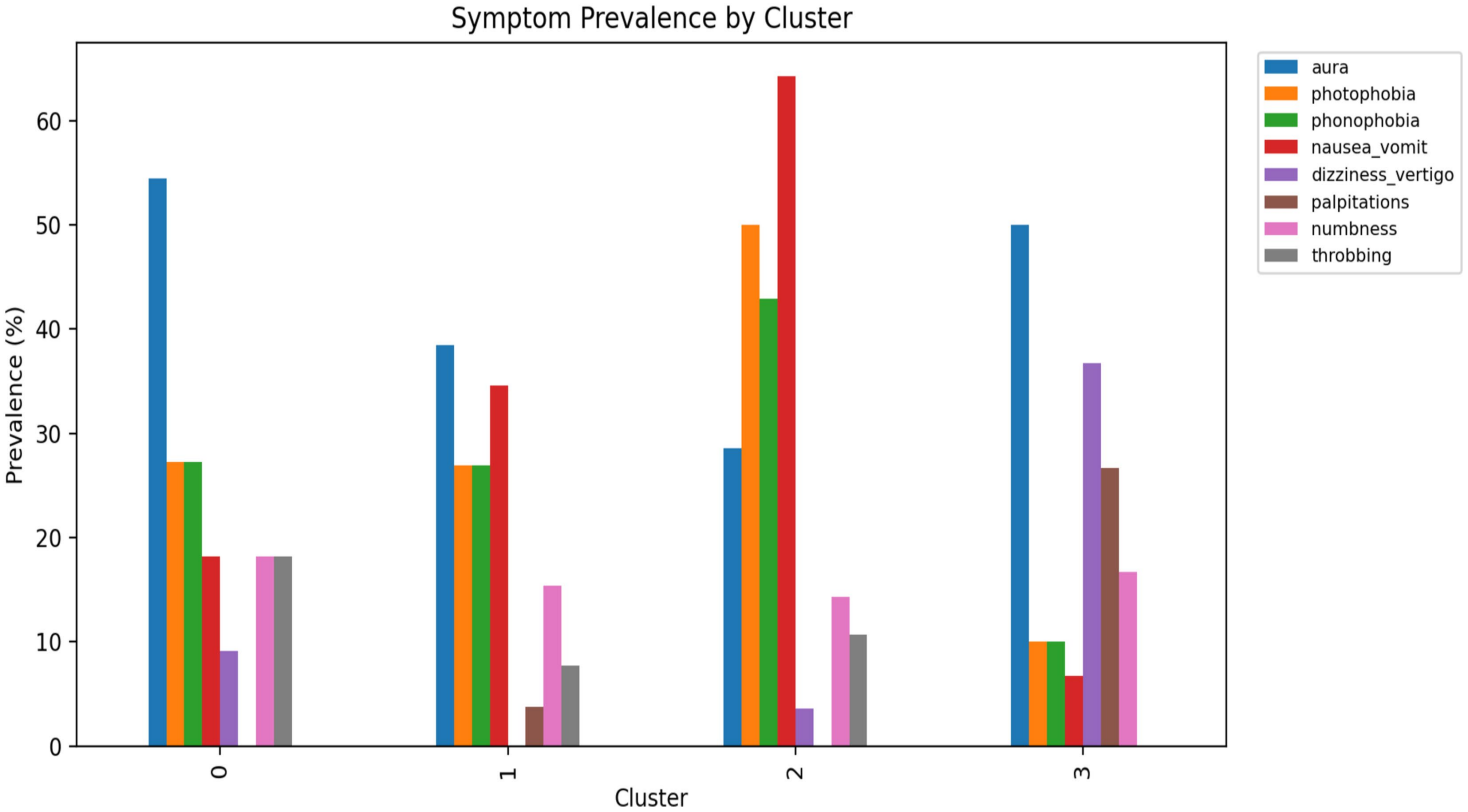
Symptom prevalence (%) by cluster, illustrating distinct autonomic signatures across phenotypes.

**Figure 4:**
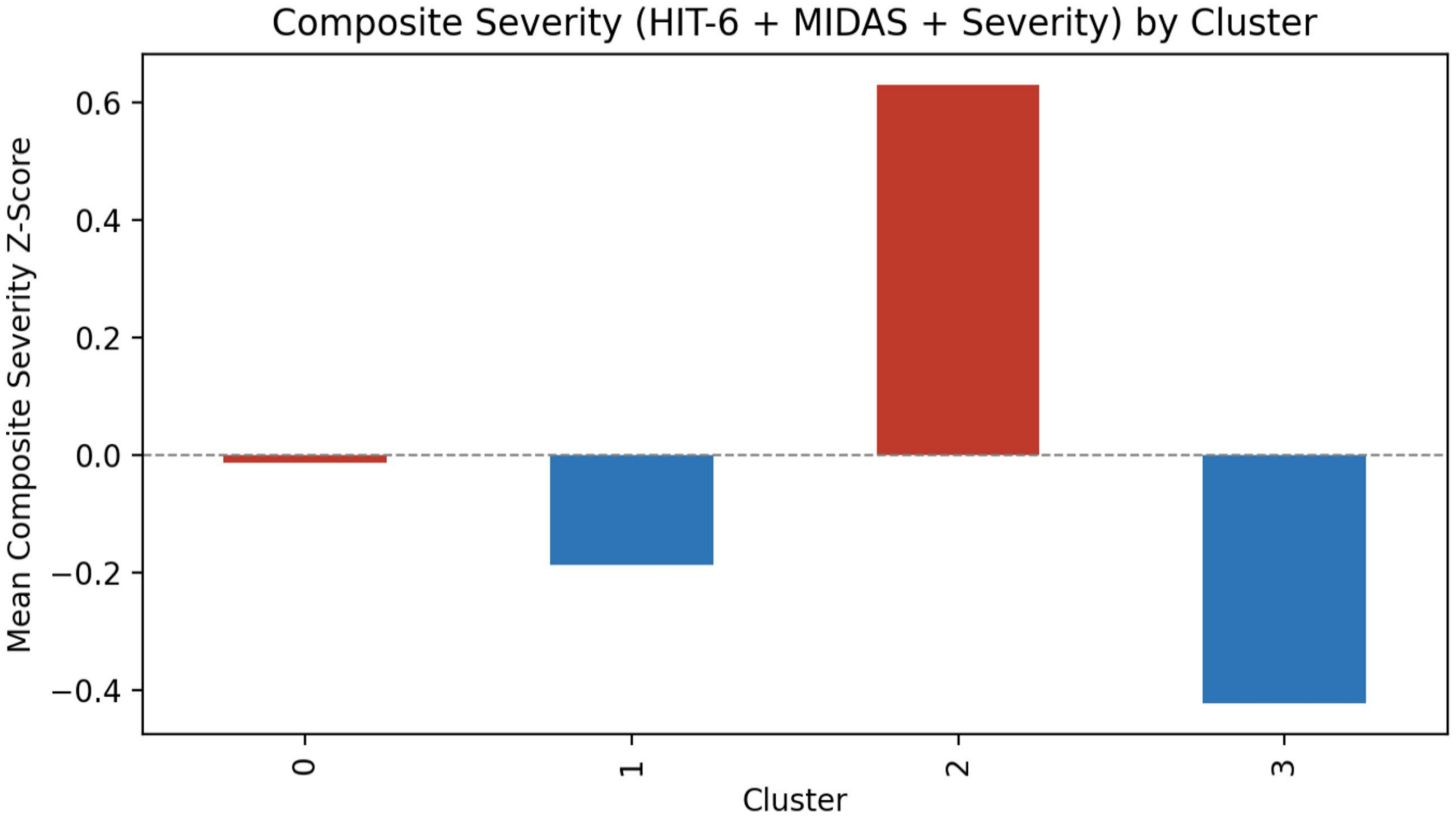
Composite severity z-score by cluster. Red bars indicate the severe phenotype (clusters 2 and 0).

To remove the clustering step entirely, we related serum vitamin D to a continuous composite severity score (the mean of standardized HIT-6, MIDAS, and attack severity). Vitamin D was not correlated with composite severity (Spearman *ρ* = 0.15, *p* = 0.14) and was not associated with it in a multivariable linear model adjusting for age, sex, and duration (*β* = 0.005 per ng/mL, *p* = 0.58). In the same model, female sex was independently associated with greater severity (β = 0.37, *p* = 0.03). These cluster-independent results reproduce both principal findings of the clustered analysis, the vitamin D null and the female-sex effect, demonstrating that neither conclusion is an artifact of an unstable partition. The full robustness results are reported in Supplementary Table S2 (see Figures 3, 4).

## 4 Discussion

This study identified four clinically interpretable migraine phenotypes from routine outpatient data and examined whether serum vitamin D differed across these subgroups. While vitamin D deficiency was strikingly prevalent across the entire cohort, with two-thirds of patients falling below 20 ng/mL, formal statistical testing did not demonstrate significant differences across the derived phenotypes. Nonetheless, one aura-rich, left-sided subgroup exhibited particularly low levels, and the findings offer insights into both migraine heterogeneity and the vitamin D–migraine relationship that merit further investigation (13).

The four phenotypes share conceptual similarities with those reported in prior clustering work. Woldeamanuel et al. (4), who applied cluster analysis to 150 chronic migraine patients, found subgroups differing in disability, psychological comorbidity, and medication overuse. The present study extends this by incorporating pain topography and autonomic features, both of which proved to be strong discriminating variables. A network analysis study by Shang et al. (14) similarly found that non-headache symptom patterns, including vestibular and autonomic features, revealed distinct migraine subgroups, reinforcing the value of moving beyond headache frequency alone in classification.

The high-disability cluster (Cluster 2) is noteworthy for its rich autonomic symptom burden: over 90% reported at least one autonomic symptom, with nausea/vomiting (64.3%) and photophobia (50.0%) as dominant features. This cluster was overwhelmingly female (85.7%), consistent with evidence that women experience more severe migraine-related disability and richer associated symptomatology. The GBD 2023 headache analysis confirmed that disability attributable to migraine is more than double in females compared with males (2). Our logistic regression supported this observation: female sex was the only significant predictor of severe phenotype membership (OR 3.37, *p* = 0.021), consistent with the well-established female predominance in migraine disability (15).

The prevalence of deficiency in our sample (67.4% below 20 ng/mL) is broadly concordant with other migraine populations. Song et al. (6) reported 77.1% of migraineurs below this threshold, and a meta-analysis by Das et al. (16) found mean 25-hydroxyvitamin D levels significantly lower in migraineurs than non-migraine controls (mean difference −4.44 ng/mL, 95% CI − 6.11 to −2.77). The lack of a significant between-cluster difference, however, suggests that the vitamin D–migraine association may operate more uniformly across phenotypes rather than concentrating in the most severe subgroup, as we had hypothesized.

The notably low vitamin D in Cluster 0 (median 9.54 ng/mL) warrants attention. This aura-rich subgroup fell in the severely deficient range. An association between vitamin D deficiency and aura has biological plausibility: vitamin D modulates cortical excitability through effects on calcium channels and glutamate signaling (17), and cortical spreading depression is sensitive to excitability changes. Patel et al. (18), in a nationwide retrospective study of 446,446 migraine hospitalizations, found that vitamin D deficiency was significantly associated with migraine-related loss of function (OR 3.12, 95% CI 2.38–4.08). Although our study lacked power for this small subgroup, the pattern invites investigation in aura-enriched populations.

The non-significant regression finding aligns with more cautious assessments. Lippi et al. (19) found no convincing evidence of a specific vitamin D–migraine link, noting deficiency rates among migraineurs did not differ from population norms. More recently, however, a Mendelian randomization study by Yin et al. (20) provided genetic evidence for a modest causal protective effect of higher vitamin D levels on migraine risk, suggesting the relationship may be genuine but small in effect size, consistent with our study being underpowered to detect such differences across subgroups.

The identification of Cluster 3, distinguished by dizziness/vertigo and palpitations rather than traditional sensory symptoms, suggests the existence of an “autonomic-predominant” phenotype with lower disability. Similar autonomic clustering has been described in vestibular migraine populations (17). This cluster also had a balanced sex ratio (50% female), contrasting with other clusters and potentially pointing to distinct pathophysiological underpinnings. Vitamin D supplementation trials have not stratified by autonomic profile, and future trials might consider doing so.

A meta-analysis of six RCTs by Hu et al. (8) reported that vitamin D supplementation reduced monthly headache attacks (MD − 2.74, 95% CI − 3.82 to −1.67) and MIDAS scores. Combined with the widespread deficiency observed across all phenotypes in our study, these findings suggest that vitamin D screening and repletion may be a pragmatic, low-risk adjunctive intervention regardless of clinical subtype (21).

### 4.1 Limitations

Several limitations should be acknowledged. The sample size (*N* = 95) limits power, particularly for detecting modest between-cluster vitamin D differences with four groups. The low silhouette score (0.168) indicates substantial cluster overlap, and the phenotype boundaries should be regarded as exploratory (22). Symptom data were extracted from free text, introducing potential underascertainment; symptoms such as osmophobia and premonitory features were not systematically captured. The cross-sectional design precludes causal inference (23). Potential confounders of vitamin D status including body mass index, sun exposure, dietary intake, and supplementation use were unavailable. Additionally, this study was conducted in Saudi Arabia, where population-level vitamin D deficiency prevalence ranges from 40 to 90%, likely compressing between-group variance and reducing our ability to detect phenotype-specific differences. Two further limitations merit explicit discussion because they bear directly on interpretation. First, the study did not include a concurrent non-migraine control group, and it therefore cannot establish whether vitamin D deficiency is specific to migraine. We regard this as a boundary on the question the study can answer rather than a flaw in its execution: the aim was within-disease phenotypic stratification, not case control etiology. Consistent with this, the cohort’s deficiency prevalence (67.4% < 20 ng/mL) was statistically indistinguishable from contemporary Saudi general-population estimates of ~63–65% (binomial *p* = 0.40) (11, 12), and 25(OH)D did not differ by sex within the cohort. The most parsimonious interpretation is that the deficiency observed here reflects the very high regional background rate rather than a migraine-specific phenomenon precisely the scenario that an absent control group cannot exclude, and which we therefore state explicitly. The case–control question has been addressed in dedicated studies and meta-analyses, which report a small mean reduction in migraineurs (e.g., −4.44 ng/mL) (16); our data neither confirm nor refute that small effect and were not designed to. Second, the clusters are statistically unstable: bootstrap resampling yielded a mean Jaccard coefficient of 0.48, below the 0.60 threshold conventionally taken to denote a reproducible cluster, so the four phenotypes should be read as a descriptive lens on a continuous phenotypic spectrum rather than as discrete latent subtypes. To guard against this instability propagating into the conclusions, we confirmed that the central findings hold under alternative mixed-data algorithms (k-prototypes, Gower-based hierarchical clustering) and, more decisively, in cluster-free continuous analyses that bypass partitioning altogether (Section 3.6). The vitamin D null and the female-sex effect were reproduced in every case, indicating that the substantive message does not rest on the specific partition. Finally, one patient aged 8 years was included in the primary analysis. Because pediatric migraine may differ clinically from adult migraine, we conducted a sensitivity analysis excluding this patient (*N* = 94). All cluster assignments remained unchanged, and key statistical tests yielded similar results: Kruskal–Wallis H = 5.19, *p* = 0.158; logistic regression OR for vitamin D = 1.00 (95% CI 0.95–1.05, *p* = 0.975); female sex OR = 3.42 (95% CI 1.21–9.67, *p* = 0.020). Full results are reported in Supplementary Table S1.

## 5 Conclusion

This exploratory study demonstrates that data-driven cluster analysis of routine clinical features can identify interpretable migraine phenotypes, which are best understood as a descriptive partition of a continuous clinical spectrum rather than discrete subtypes. Vitamin D deficiency was highly prevalent across the entire cohort but did not differ significantly between phenotypes, a null that proved robust to the clustering algorithm and to cluster-free analysis, and matched the regional background rate, suggesting it reflects population-level deficiency rather than a migraine-specific signal, suggesting that screening may be broadly applicable. The particularly low vitamin D in an aura-rich subgroup is hypothesis-generating and warrants confirmation in larger, prospective studies that can adjust for the many confounders of vitamin D status and that incorporate non-migraine control groups to separate disease-specific from background-population effects.

## Data Availability

The original contributions presented in the study are included in the article/supplementary material, further inquiries can be directed to the corresponding author.
